# Inter-rater Variability in Malaria Microscopy at the LEKMA Hospital, Ghana

**DOI:** 10.1155/2020/8897337

**Published:** 2020-12-09

**Authors:** Andrew Nii Adzei Bekoe, Emmanuel Alote Allotey, Elliot Elikplim Akorsu, Albert Abaka-Yawson, Samuel Adusei, Godsway Edem Kpene, Precious Kwablah Kwadzokpui

**Affiliations:** ^1^Department of Medical Laboratory Sciences, School of Allied Health Sciences, University of Health and Allied Sciences, Ho, Ghana; ^2^Laboratory Department, Ledzokuku Krowor Municipal Assembly (LEKMA) Hospital, Accra, Ghana; ^3^Department of Obstetrics and Gynaecology, University of Cape Town, Cape Town, South Africa

## Abstract

**Background:**

Malaria remains a major cause of morbidity and mortality worldwide and particularly in sub-Saharan Africa where it is endemic. As such, it is important that a proper diagnosis is made before treatment is initiated. Malaria parasite count plays a key role in the diagnosis and management of malaria. Variations in ratings by laboratory personnel can impact negatively on the treatment regimen for malaria-infected patients. The study is thus aimed at evaluating and comparing the proficiency and parasitaemia counts by two different categories of laboratory staff at the LEKMA Hospital, Ghana.

**Materials and Methods:**

A total of 200 confirmed malaria-positive samples were used in the study. Six thick and thin films were prepared from each sample and uniquely labelled. Two of the six slides were given to two WHO-accredited malaria microscopists to examine and report their respective parasite count/*μ*l (parasite count/WBC × 8000). These were used as the reference for the two categories of laboratory staffs: rater A being diploma holders (Technical Officers referred to as untrained rater) and rater B being degree holders (Medical Laboratory Scientist referred to as trained rater) at the LEKMA Hospital.

**Results:**

In comparison to the expected outcome, the parasite count by the rater group A (190 (151-239)]) and the rater group B (177 (140-224)) demonstrated significant positive correlation (*r* = 0.995, *p* < 0.0001 vs. *r* = 0.995, *p* < 0.0001, respectively) with the expected outcome in the cases of heavy parasitaemia. A statistically significant difference (*p* < 0.05) between counts by the different raters in low parasitemia was observed in this study. A persistent nosedive inter-rater agreement from *k* = 0.82 to *k* = 0.40 with increasing density cutoff was observed in this study.

**Conclusion:**

The study observed that the degree of inter-rater agreement of parasite density count by various categories of laboratory personnel is almost perfect. However, the parasite count between raters varied significantly with very low levels of parasitemia but better correlated with heavy parasitemia.

## 1. Introduction

Malaria remains a major cause of morbidities and mortalities worldwide. Annually, an estimated 3.2 billion and 429,000 morbidities and mortalities occur, respectively [1]. Sadly, the majority of the cases occur in sub-Saharan Africa where it is reported that a child dies of malaria every minute with increased risk of stunting [1–3]. The diagnosis of malaria over the years has been based on parasite identification and subjective quantification by laboratory personnel using the plus (+) system [4]. However, in recent years, it has become an established practice to count the parasites present alongside white blood cells or parasitized red blood cells in stained blood film [5, 6]. This gives a better diagnosis and aids in follow-up after the treatment has commenced. It is reported that thick blood film has a sensitivity exceeding 80% when parasitaemia is above ten parasites per microliter of blood [5]. Furthermore, microscopy provides a quantitative assessment of parasitaemia and parasite stages in peripheral blood [5]. Due to the importance placed on the microscopic diagnosis of malaria, it forms the basis for most diagnostic decisions in most hospitals. In Ghana, as in many other African countries, the standard diagnostic technique for malaria is the microscopic examination of stained blood slides [1]. As malaria prevalence increases in many African countries, including Ghana, the ability to identify cases of malaria parasitaemia has become increasingly important [7]. Good quality microscopy conducted by skilled medical laboratory scientists for correct identification of parasites and the ability to manage appropriate quality control are among the key requirements in the management of malaria [1, 8]. Secondly, parasitaemia-related variable in epidemiological studies is parasite density, expressed as parasites/*μ*l of blood. This measure has recently gained importance in the context of the definition of clinical malaria episodes either in studies on the development of naturally acquired immunity [9] or intervention studies looking at the efficacy of insecticide-treated materials [10, 11] or antimalaria vaccines [12, 13]. As regards variations that may occur among individual ratings, a study by O'Meara et al. [14] observed that variations in parasite count reported by raters were due to sample handling or random distribution of parasites in the various blood samples. Another study reported this variance could be due to both systematic errors which include but not limited to human error, excessive or inadequate blood on a slide, poor slide preparation, staining problems, and parasites being obscured or difficult to identify in thick films [5]. Most of the studies in the country focused on the diagnostic methods rather than the personnel rating capabilities. For instance, Osei-Yeboah et al. [2] compared the diagnostic ability of RDT and microscopy. With little data on the inter-rater variability for determining parasite density, this study sought to compare the proficiencies of two different categories of laboratory staffs at the LEKMA Hospital, Ghana.

## 2. Materials and Methods

### 2.1. Malaria-Positive Slide Preparation

A total of 200 confirmed malaria-positive samples were used in the study. Six slides were prepared by qualified laboratory scientists in the haematology unit of the laboratory who possess the prerequisite knowledge in blood film preparation from each of the samples that were selected. Following standard operating procedures, both thick and thin blood films were prepared using 6 *μ*l and 2 *μ*l of blood, respectively, on the same slides for purposes of convenience during microscopic observation. The slides were allowed to air-dry properly after which the thin film of each slide was fixed in absolute methanol. This was done by placing the slide on a staining rack, and then, small drops of absolute methanol were added to the thin film by the use of Pasteur pipettes making sure that the alcohol did not splash or spill over onto the thick film. The slides following fixation in absolute methanol were allowed to air-dry for the second time and subsequently subjected to 10% Romanowsky Giemsa staining technique for 10 minutes as outlined in Monica Cheesbrough, Part 1 [15]. The slides were washed after the 10-minute staining by gently flooding the slide with clean tap water so as to prevent the washing away of the films. The slides were subsequently air-dried properly, ready for microscopy.

### 2.2. Rater Selection

Six thick and thin blood films were prepared from each sample and uniquely labelled. Two of the six slides were given to two WHO-accredited malaria microscopists (each with competence level 1) to examine and report their respective parasite count/*μ*l (parasite count/WBC × 8000); their results served as the yardstick for comparison, i.e., the expected outcome with the two categories of laboratory staffs. Raters categorized as rater A were diploma holders (Technical Officers referred to as untrained staff) whilst rater B were degree holders (Medical Laboratory Scientists referred to as trained staff); all are from the LEKMA hospital. The remaining four slides were randomly distributed to raters, i.e., the laboratory staffs, to examine and report parasite counts. 100 of these slides were examined by each rater category. For each category of raters, i.e., rater A and rater B, the first 50 slides were examined by those who had received malaria refresher training programme and their competency levels assessed a month prior to this study in addition to basic malaria training and the other 50 by those without the malaria refresher training program, however, had basic laboratory training. Malaria parasites were identified and counted together with white blood cells. Each rater was allowed to read and score between 30 and 40 slides per day as recommended by the WHO. Each rater was allowed a maximum of 20 minutes to determine the WBC count, parasite count, and parasite density.

### 2.3. Parasite Count Technique

The raters were permitted to use either the thick or thin films and to count parasites per white blood cells (WBCs) or red blood cells (RBCs), respectively. There were no guidelines given as to how many WBCs should be indexed when measuring the parasite density. The entire blood film was examined using oil immersion objective. Starting at the top left of the smear, a typical field with both parasites and white cells were counted using an assigned key on the tally counter for each parasite or white cell. The battlement movement method was used in the counting process from one field to the other.

### 2.4. Data Handling and Statistical Analysis

Statistical analysis was performed using GraphPad Prism 6.0 (GraphPad Software, San Diego California, USA). The parasite densities of the two microscopic rater groups and the expected parasite densities (provided by WHO-accredited microscopists) were compared using the Student's *t*-test and ANOVA, respectfully. The Kappa index [16] was used to assess the inter-rater agreement of categorical data while plotting of the difference of the two measurements against their mean and calculation of the mean difference and “limits of agreement” (mean ± standard deviations) was used for continuous data as suggested by Bland and Altman (1986). All analyses on parasite densities were performed after log transformation of the data.

### 2.5. Ethical Consideration

Ethical clearance was sought from the Research Ethics Committee of the University of Health and Allied Sciences and also from the management of the LEKMA Hospital to use routine samples that came to the laboratory for the study. Confidentiality of patients' records was ensured, and the resulting data are used for academic purposes only.

## 3. Results

The study observed that the mean WBC count by the two rater categories A and B were significantly lower (<0.0001) compared to the expected WBC. However, the mean parasite count and parasite density were comparable to the expected (*p* > 0.05) as shown in Table 1.

Table 2 presents the correlation results and mean percentage error difference in log-transformed parasite densities. There was a significant positive correlation observed between the two rater categories as well as the interrating. However, there was a higher but statistically comparable mean difference between rater B (0.141) and the expected compared to that of rater A (0.037) and the expected. The variation observed among the rater B was very minimal compared to that of the rater A category.

**Figure 1:**
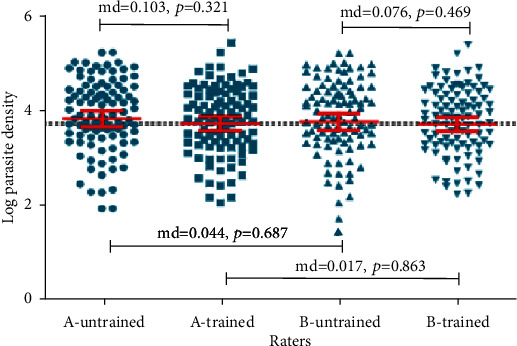
Trained and untrained rater difference in parasite density/*μ*l.

Figure 1 shows the log parasite density observed by trained and untrained raters. The results show that the parasite density observed by untrained rater A was relatively higher but not significantly different compared to trained rater A with a mean difference of 0.103 (*p* = 0.321). The limits of agreement were calculated as 1.87 to 2.07. The antilog of these figures gives 0.15 and 7.92, indicating that the density count of the trained rater A was 0.15–8.0 times that of the untrained rater A. Similarly, the parasite density observed by untrained rater B was higher than that of the trained rater B with a mean difference of 0.076 (*p* = 0.469). The limits of agreement were calculated as -1.93 to 2.08 the antilog of which gives 0.15 and 8.0, indicating that the density count of the trained rater B was 0.15–8.0 times that of the untrained rater B. In addition, parasite density observed by untrained rater A was higher than that by the untrained Rater B with a mean difference of 0.044 (*p* = 0.687) with limits of agreement as -0.135 to 0.223. The antilog gives 0.87 and 1.26, indicating that the density count of the untrained rater A was 0.9–1 times that of the untrained rater B. Similarly, the parasite density observed by trained rater A was higher than that by the trained rater B with a mean difference of 0.017 (*p* = 863) with limits of agreement as -0.111 to 0.145. The antilog gives 0.89 and 1.16, indicating that the density count of the trained rater A was 0.9–1.0 times that of the trained rater B. The parasite densities observed by the untrained raters were higher than the expected counts, and the parasite densities of the trained raters were lower than expected; however, the mean differences were not significant.

From Table 3, the results show similar outcomes for low parasitaemia with increased discrepancies as the parasite density increases. However, rater B is in close agreement with the expected outcome for the parasite density. The inter-rater agreement continuously decreased from *k* = 0.82 to *k* = 0.40 with increasing density cutoff. Variability was shown to be wide at higher densities for rater A and somewhat close for rater B.

**Figure 2:**
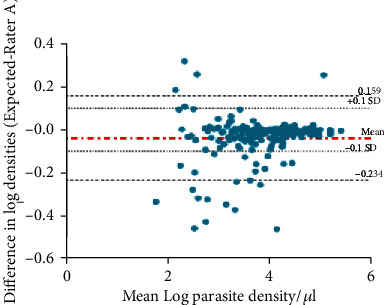
Bland-Altman plot demonstrating the agreement between the expected parasite density and parasite density from rater A (parasite density values are log-transformed).

Figure 2 shows the Bland-Altman plot demonstrating the agreement between expected parasite density and that for rater A. The results show a mean difference (bias) of 0.037. The limits of agreement were calculated as -0.234 to 0.159, thus indicating that the density count of rater A was 0.79–1.17 times that of the expected count.

**Figure 3:**
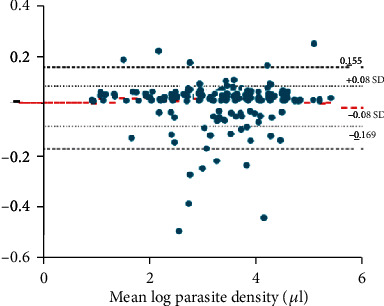
Bland-Altman plot demonstrating the agreement between expected parasite density and parasite density from rater B (parasite density values are log-transformed).

Figure 3 shows the Bland-Altman plot demonstrating the agreement between the expected parasite density and parasite density of rater B. The results show a mean difference (bias) of 0.141. The limits of agreement were calculated as -0.169 to 0.155; thus, the density count of rater B was 0.84–1.17 times that of the expected count.

**Figure 4:**
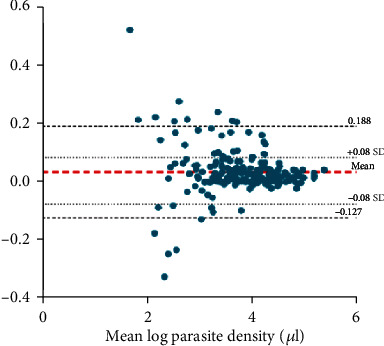
Bland-Altman plot demonstrating the agreement between parasite density of rater A and B (parasite density values are log-transformed).

Figure 4 shows the Bland-Altman plot demonstrating the agreement between the expected parasite density and parasite density of rater B. The results show a mean difference (bias) of 0.141. The limits of agreement were calculated as -0.127 to 0.188 indicating that the density count of rater A was 0.88–1.2 times that of rater B.

## 4. Discussion

The results of this study indicate that there was an almost perfect agreement in the ratings made by the different categories of raters as reflected by the correlation of between 0.995 and 0.997 (*p* < 0.0001) [17]. The observed high level of agreement could partly be a reflection of the efforts made so far in equipping the laboratories and capacity strengthening for the technologists involved in malaria microscopic diagnosis in Ghana. When the two parasite counts for each Rater were compared, a considerable variability was found with one rating being 0.88–1.20 times the other although variability was not significant contrary to the observations of Kilian et al. [18]. This variability also differs from that found in two other studies which used the same statistical approach but compared two methods of enumerating parasite densities [19, 20]. Furthermore, the study found a considerable variability in the parasite count of the trained and untrained raters where the ratings of untrained rater A was 0.15–8 times higher than those of trained rater A. The variability of the parasite density generated by the trained rater group was statistically comparable to the expected parasite density, implicating the level of training as an impactful contributing factor in the accurate malaria parasitemia diagnostic capability of the laboratory technologist/scientists. As clearly indicated by Billo et al. [21], substantiating the findings of this study, the level of training among other factors such as the quality of slide preparation, quality of reagents and microscopes, random selection of microscope fields, motivation, and concentration/focus during microscopy all influences the outcomes of the variability of the parasite density. A few studies have shown that the malaria prevalence, estimated as the proportion of stained blood slides found to have malaria parasites, tends to vary considerably at low parasite densities ranging between 4 and 100/*μ*l of blood, but much less so above that density [7, 21]. This was however contrary to the finding of this study as malaria parasitaemia varied with increasing density indicated by the decrease in kappa index. Tangpukdee et al. [22] contended that malaria microscopist technologists located in the malaria endemic areas are faced with large numbers of blood slides to read daily, which tend to decrease their ability to perform accurately and with precision when observing slides with very high parasite count.

## 5. Conclusion

The duration of training in school and continuous refresher training in malaria microscopy has an impact on proper microscopic detection and malaria parasite density estimations. It is asserted that consented plans and efforts made to improve microscopic diagnosis in Ghana through regular trainings of technologists, the supply of equipment and reagents within the means available, and engaging in corrective supervision of health facilities have impacted malaria diagnosis positively.

## 6. Limitations

Due to the short period of the study, a larger sample size could not be used as the majority of the samples brought for investigation proved to be negative for malaria. Also, only a few WHO-accredited malaria microscopists and technical laboratory staff were involved in the study.

## 7. Recommendations

Given that microscopy is still the mainstay and the gold standard for the diagnosis, more resources will need to be available to improve further and sustain the current level of accuracy in order to save more lives and thus build more trust between the health care system and the patients. It is therefore highly recommended that the frequent Malaria Diagnosis Refresher training workshops be organised to help reorient medical laboratory personnel towards effective diagnosis. The resources and logistics as well as consumables needed for effective diagnosis should be available as much as possible to ensure that effective diagnosis can always be carried out.

## Figures and Tables

**Table 1:** Rater difference in WBC, parasite count, and parasite density.

Parameter	Ratter A mean (95% CI)	Ratter B mean (95% CI)	Expected outcome mean (95% CI)	*p* value
WBC	212.3 (209-215)^∗^	212.4 (209-215)^∗^	219.7 (215-224)	<0.0001
Parasite count	190 (151-239)	177 (140-224)	180 (143-228)	0.8920
Parasite density/*μ*l	7151 (5658-9039)	6664 (5254-8453)	6565 (5158-8355)	0.8700

^∗^Significantly different from the expected outcome at *p* < 0.05.

**Table 2:** Pearson's correlation coefficient and mean percentage error difference in log parasite densities.

Parameters	Mean difference (parasites/*μ*l)	*t*-test *p*value	*r*	*p* value	%Δ
Expected vs. rater A	0.037	0.612	0.995	<0.0001	1.0
Expected vs. rater B	0.141	0.931	0.995	<0.0001	0.1
Rater A vs. rater B	0.031	0.680	0.997	<0.0001	0.8

*r*: Pearson's correlation; %Δ: percent change; *p* value significant at <0.05.

**Table 3:** Sample estimates and inter-rater agreement (Kappa index) for the prevalence of parasitaemia above a specific density cutoff comparing two rater microscopy parasite density counts.

Cut-off density/*μ*l	Rater A	Rater B	Expected	Kappa index
	(%)	(%)	(%)	
1000	14.0	14.5	15.0	0.82
2500	12.0	13.0	13.0	0.77
5000	13.0	14.5	14.5	0.69
10000	15.0	14.5	13.5	0.63
20000	13.5	14.0	15.0	0.58
30000	32.5	29.5	29.0	0.40

## Data Availability

The data used to support our findings are available on reasonable request.
